# Short-Duration Systemic Lidocaine for the Management of Refractory Chronic Pain in Pediatrics

**DOI:** 10.3390/children12101349

**Published:** 2025-10-08

**Authors:** Bobbie Riley, Christine Shusterman, Teresa O’Neil, Carolina Donado, Kimberly Lobo, Anjali Koka, Sarah Nelson, Monique Ribeiro, Pradeep Dinakar, Jean Solodiuk, Neil Schechter, Christine Greco

**Affiliations:** 1 Department of Anesthesiology, Critical Care and Pain Medicine, Boston Children’s Hospital, 300 Longwood Avenue, Boston, MA 02115, USA; christine.shusterman@childrens.harvard.edu (C.S.); kimberly.lobo@childrens.harvard.edu (K.L.); sarah.nelson@childrens.harvard.edu (S.N.);; 2 Department of Anaesthesia, Harvard Medical School, Boston, MA 02115, USA; 3 Department of Psychiatry and Behavioral Sciences, Boston Children’s Hospital, Boston, MA 02115, USA; 4 Department of Psychiatry, Harvard Medical School, Boston, MA 02115, USA

**Keywords:** lidocaine, chronic pain, pediatrics

## Abstract

**Objectives:** Multidisciplinary management of chronic pain benefits many patients, although some continue to experience refractory pain. Administration of lidocaine infusions (LI) to manage certain chronic pain conditions has been reported in adults, but evidence is limited for its utility in managing pediatric chronic pain. We reviewed LIs for refractory pain in children to (1) describe the patient population that received LI and (2) evaluate the response to LI. **Methods:** With IRB approval, a retrospective review of patients receiving LI for refractory pain between 2016 and 2021 was conducted at Boston Children’s Hospital. Demographic, medical, pain, sleep, and school function information was collected through self-report and from the electronic medical record. Longitudinal outcomes for a subset of these patients were analyzed using the Chronic Pain Repository database. **Results:** During the study period, 3959 patients presented for management of chronic pain. Among this population, 184 (5%) patients aged 22 years or younger ultimately received LI as part of their pain management. A total of 350 LIs were administered and were well tolerated. During approximately 42% of the infusions, patients experienced an immediate statistically significant decrease in pain scores. Among the patients with follow-up data, pain improvement was not sustained. **Discussion:** LI for the treatment of chronic pain appears safe and may be useful for managing refractory pain in pediatrics. Although approximately half of the patients who received LI reported an immediate positive response, the small follow-up sample did not show a continued response. Study limitations preclude demonstrating long-term efficacy of LI; therefore, a prospective study is critical.

## 1. Introduction

Although the multidisciplinary management of chronic pain with cognitive-behavioral therapy, physical therapy, and medical interventions is beneficial for many children and adolescents, some patients continue to experience refractory pain [1]. In a limited number of studies, lidocaine infusions have demonstrated possible benefits in the management of chronic pain [2,3,4]. Lidocaine exerts its known effect via sodium channel blockade. Its mechanism for the treatment of chronic pain conditions is not entirely understood, although it is suggested to be independent of conduction blockade [5]. Some proposed mechanisms for long-lasting analgesia from lidocaine include modulation of neurotransmission and a potential anti-inflammatory effect [2,4,6].

Lidocaine infusions (LI) have been used in the management of both acute and chronic pain, with some evidence for its intraoperative use as an analgesic in the adult population [7,8]. A review among adult patients suggests that systemic lidocaine appears to be safe and may decrease the development of chronic post-surgical pain syndromes when used for the treatment of their chronic or neuropathic pain syndromes [6]. A systematic review of adult patients with cancer pain concluded that systemic lidocaine was more effective than placebo for the treatment of neuropathic pain [7,9]. There is limited information regarding the use of LI for acute and chronic pain in children. Data from retrospective series suggest that LI may be useful for the management of cancer pain and other refractory pain conditions, such as erythromelalgia and status migrainosus in children and adolescents [10,11,12,13,14].

Reports also suggest that LI is well tolerated as a component of multimodal postoperative management for pediatric patients, although with mixed results for efficacy [8,15,16]. One retrospective review of refractory headache management in adolescent patients showed that lidocaine appeared to be safe and was associated with a 50% reduction in pain scores after approximately 16 h of continuous lidocaine infusion [10]. However, there are no randomized controlled trials and only a few retrospective small series on the safety and tolerability of LI in pediatric patients [6,8,17], which makes the broader application of LI in this population unclear.

At Boston Children’s Hospital (BCH), we have administered LI in an outpatient setting for the management of refractory chronic pediatric pain conditions since 2016. We present data gathered from our 5-year experience using short-duration LIs for refractory chronic pain in children and adolescents. We describe the population who received LI, their tolerance, and, in a limited subsample, their long-term response. We hypothesized that systemic lidocaine is safe and associated with decreased pain scores and improvements in physical and psychosocial factors in our study population.

## 2. Materials and Methods

The current investigation was approved by the institutional review board at BCH (IRB-P00035746) with a waiver of consent for a retrospective review. Patients were initially seen at the hospital’s Multidisciplinary Pain Clinic (MPC). Infusions occurred at BCH, a 404-bed quaternary pediatric medical center, in a procedural unit. We performed a retrospective chart review of pediatric patients receiving LI for pain between 1 May 2016 and 30 June 2021 to describe the population and patient response to LI. We compared demographic data of those receiving LI with those who did not.

We used the following data sources: the electronic medical record (EMR), as well as the Chronic Pain Data Repository (CPDR). The EMR includes structured and unstructured data, including socio-demographics, vital signs, laboratory and test results, prescribed medications, billing codes, and clinical notes for services provided. The CPDR is a separate database that collects patient-reported outcomes and is maintained by the MPC. This database can be accessed under a standardized research protocol approved by the hospital’s institutional review board (IRB) in October 2018 [18]. Data collected through the CPDR include information about pain, child development, and medical history, as well as physical, sleep, and psychological functioning. This information is collected via two electronic questionnaires (one with demographic and pain characteristics and the other with standardized psychological questionnaires) sent to families and completed prior to the patient’s initial encounter with the MPC. Using the same platform, as a pilot quality improvement initiative for patients who received LI between 2019 and 2020, we sent subsequent electronic questionnaires to collect post-infusion pain scores, sleep, and physical and psychological functional outcomes. These electronic questionnaires were sent on the day of the LI and then following each LI at 2 weeks, 4 weeks, and monthly intervals thereafter.

### 2.1. Participants

We identified all medical encounters for patients who received LI using Current Procedural Terminology (CPT) codes (96365, 96366, J2001, 99215, F99215) and LI medication orders. Using the CPDR, the above-described outcomes were collected at the initial presentation of all patients seen at MPC during the study timeframe (May 2016 to June 2021). Patients older than 22 years of age were excluded from the sample.

### 2.2. Demographic Information

We collected self-reported information on basic demographics, medical history, health utilization, sleep, and school functioning. Using International Classification Codes (ICD-9 and ICD-10), we collected pain diagnoses at the first visit and with each LI. All pain-related diagnoses were then grouped into four categories of pain: musculoskeletal, neuropathic, headache, and abdominal. Information on psychological comorbidities (i.e., anxiety or depression) was also collected using ICD codes.

### 2.3. Lidocaine Infusion

In 2016, the MPC developed an LI guideline based on available evidence and clinical experience. LIs are offered to patients who do not respond to the conventional multidisciplinary treatment plans, including medications, physical therapy, and cognitive-behavioral therapy. The LI guideline includes dosing and monitoring requirements, as well as inclusion and exclusion criteria. Patients with a history of cardiac, liver, or kidney disease, uncontrolled seizures, or allergies to amide local anesthetics are not eligible to receive LI due to increased risk. Routine screening laboratory studies are not performed. The guideline was approved by the hospital’s Pharmacy and Therapeutics committee prior to implementation. Table 1 shows our current selection criteria. Table 2 shows LI dosing. All LIs occurred in the procedural unit within the hospital; a pain clinician is always available for the duration of the LI, and intra-lipid infusion is available if concerns of local anesthetic severe toxicity (LAST) arise. No lidocaine levels are measured as part of routine care for the short-duration LI.

### 2.4. Tolerability of the Infusion

Vital signs were assessed continuously throughout the LI per guidelines. Hemodynamic changes were considered sustained if they lasted 5 min or more. Mild tachycardia and hypertension were defined as a sustained increase of up to 40% of the baseline value. Severe tachycardia and hypertension were defined as a sustained increase of 40% or more of the baseline value. Severe bradycardia was defined as a decrease in the heart rate of more than 40% of the baseline value or an absolute value of 35 or fewer beats per minute. Hypotension was defined as a decrease of 40% or more of the baseline value. Patients with possible side effects were identified using the baseline, minimum, and maximum values of all vital signs collected. Following this, a manual chart review confirmed or refuted the presence of adverse effects. The presence of additional adverse effects such as nausea, dizziness, and a metallic taste were identified by nursing documentation in the EMR.

### 2.5. Pain Scores

All documented pain scores during the LI were collected and timestamped. All scores were on a 0–10 scale. Most scores (92%) were recorded using the Numeric Rating Scale (NRS). Other scales used were the Face, Legs, Activity, Cry Consolability) (FLACC) scale, the Wong–Baker FACES scale, and the Individualized Numeric Rating Scale (INRS). The maximum pain score was used if two or more pain scores were collected at the same timestamp. To ensure we were measuring a clinically significant reduction in pre-infusion pain scores, we defined positive effect as ≥1 point reduction for patients with pre-infusion pain scores of <4/10 and a ≥2 point reduction for patients with pre-infusion pain scores ≥ 4.

### 2.6. Psychological Questionnaires

As part of the initial evaluation by the MPC, the following self-report measures of psychological functioning were collected:

- Functional Disability Inventory (FDI): The FDI is a validated 15-item self-report questionnaire examining one’s ability to complete physical tasks. The FDI has been validated for use in pediatric chronic widespread pain [19].
- Pain Catastrophizing Scale for Children (PCS-C) [20] is a validated 13-item self-report measure of negative thoughts and behaviors related to pain.
- Fear of Pain Questionnaire, Child Report (FOPQ-C) [21] is a 24-item measure that assesses child perceptions of pain-related fears and avoidance behaviors.
- Pediatric Quality of Life Inventory (PedsQL) [22] is a self-reported instrument that measures physical, social, emotional, and school function on a 5-point Likert rating scale. It is well-validated for use with youth with chronic pain.
- PROMIS scales are used in children ages 8 to 17. Anxiety and depression were examined using PROMIS pediatric anxiety and depression scales, short-form versions (8 items) [23,24] at baseline.

For the subset of patients who received the follow-up surveys after the LI, three questionnaires from the PROMIS pediatric profile 25 were used: anxiety, depression, and physical function mobility. Raw scores were transformed into standardized T-scores, with T-scores ≥ 60 indicating clinically significant impairment for these scales.

### 2.7. Data Analysis

Statistical analyses were performed using R (version 3.6.1) [25]. Descriptive statistics were calculated for all demographics, medical history, and psychological questionnaire variables. Comparisons between patients who received an LI and those who did not were calculated using unpaired samples t-tests. Wilcoxon signed-rank tests were used to compare median pain ratings and T-scores from psychological scales. No imputations were made to missing data.

## 3. Results

### 3.1. Population Description

Approximately 3959 patients presented to the MPC for management of chronic pain during the study period; 5% of these patients eventually received LI as part of their pain management, typically around 2 years after the initial MPC evaluation. Of the 3959 patients that were seen, 2615 had baseline data available in the CPDR. We identified a total of 350 LIs administered to 184 unique patients 22 years or younger between May 2016 and June 2021. Most of the 184 patients were female (87.0%; n = 160), with an average age of 15 years (sd = 2.5) at first visit to the MPC. The mean age of patients receiving their first LI was 17 years (sd = 2.4).

**Figure 1:**
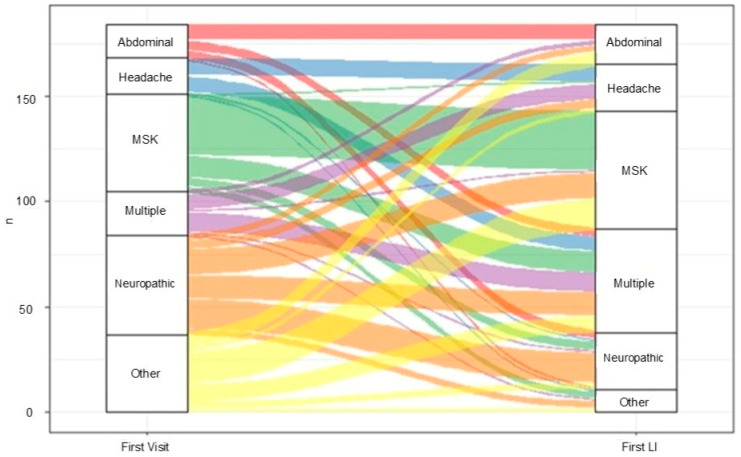
Distribution and changes of pain diagnosis between the patient’s first MPC visit and the first LI visit.

Patient pain diagnoses were categorized as musculoskeletal, neuropathic, headache, and abdominal, and were often overlapping and different between the initial visit diagnosis and the first LI diagnosis. Patient pain diagnoses at the initial visit were musculoskeletal (38.6%), neuropathic (25.5%), headache (22.3%), and abdominal pain (19.0%), with overlapping diagnoses in 21.2% of cases. Additionally, 19% had a diagnosis of anxiety or depression during the first visit. However, at the time of the first LI, the patient pain diagnoses were musculoskeletal (52.7%), neuropathic (14.7%), headache (37.0%), and abdominal (25.5%), with overlapping diagnoses in 35.5% of the cases. Diagnosis of anxiety or depression was present in 13.6% of the cases (Figure 1).

Table 3 presents the differences at initial presentation to the MPC between patients who did and did not go on to receive an LI. Of the 184 patients who received an LI, 126 (68.5%) had data in the CDPR. They reported more fatigue (*p*-value = 0.044) and disrupted sleep (*p*-value = 0.014) at baseline compared to those who did not receive LI. There were no differences in the use of assistive devices or school attendance between the patients who did and did not go on to receive LI.

Analysis of the psychological measures of patients presenting to the MPC who received and did not receive LI demonstrates that those who received an LI initially scored higher on some psychological measures. Patients who received LI reported a higher percentage of elevated PCS scores (63.2% vs. 53.2% *p*-value = 0.048) and higher raw scores in helplessness (13.7; sd = 5.6 vs. 12.4; sd = 6.4; *p*-value = 0.039) and rumination (10.9; sd = 4.0 vs. 9.8; sd = 4.7; *p*-value = 0.019) subscales when compared to those who did not go on to receive an LI. No other statistically significant differences were seen in the other psychological variables, such as FDI, PedsQL, FOP, and PROMIS scores.

### 3.2. Lidocaine Infusions

Of the 184 patients who received LI, most received a single LI (64.7%; n = 119). Approximately 16% (n = 29) received two LIs, and 19.6% (n = 36) received more than two LIs. Two patients received a maximum of 14 LIs over the 5 years (a maximum of six within a year). For those who received more than one LI, the average time between infusions was 6.4 months (sd = 6.1). The number of pain clinic visits 1 year pre and post the first LI was higher for those who received more than one LI (pre: 6.1; sd = 9.6 vs. 2.9; sd = 5.0, *p*-value = 0.004; post: 6.5; sd = 7.6 vs. 4.9; sd = 3.8, *p*-value = 0.067).

### 3.3. Events and Side Effects

Of the total 350 LIs performed, there were two separate cases concerning possible adverse effects that required further evaluation. In the first case, there was concern for an allergic reaction due to dyspnea and stridor; the LI was stopped, and following observation demonstrated hemodynamic stability and no further signs or symptoms of allergic reaction; the symptoms were felt to represent a panic episode. The second case was a patient who presented to the Emergency Department (ED) with mental status changes 20 min after discharge following their LI. This patient remained hemodynamically stable, and following evaluation in the ED, there were no concerns for local anesthetic toxicity.

The side effects reported were mild and self-limited, and included dizziness (5.4%, n = 19) and nausea (2.3%, n = 8). During 18 LIs, there were vital sign variations noted; however, upon detailed chart review, no patients became hemodynamically unstable or required discontinuation of LI because of these changes.

### 3.4. Immediate Response to LI

In 42% of the LIs (n = 146/350), there was a positive effect (≥1 point reduction for patients with pre-infusion pain scores of <4/10 and a ≥2 point reduction for patients with pre-infusion pain scores ≥4) immediately after the LI. Within the group of patients experiencing a positive effect as defined above, there was a statistically significant decrease in pain scores from 5.9 to 2.9 (*p*-value < 0.001) during the infusion. Diagnosis on the day of the LI were significantly different between those with positive effect and those without: musculoskeletal pain (35.6% vs. 23.2%, *p*-value = 0.015), neuropathic pain (19.2% vs. 16.7, *p*-value = 0.05), headache (6.2% vs. 16.3%, *p*-value = 0.004), abdominal (5.5% vs. 11.3%, *p*-value = 0.058), and multiple diagnosis (26.0% vs. 26.6%, *p*-value = 0.90). For patients who received more than one LI, we found that the percentage of patients with a positive effect increased for each consecutive LI up to the 4th one: the percentage of positive effects for the 1st LI (n = 184) was 32.6%, for the 2nd (n = 65) was 44.6%, for the 3rd (n = 36) was 50.0%, and for the 4th LI (n = 23) was 65.2%. Of the 65 patients that had a 2nd LI, 27 (42.5%) had a positive response on their first LI. Further, 66.7% (n = 18/27) of patients with a positive response to the 1st LI had a positive response to the 2nd LI vs. 28.9% (n = 11/38) of patients with no effect on the 1st LI who had a positive response to the 2nd LI (*p* = 0.006). 

### 3.5. Prospective Follow-Up

Between 2019 and 2020, patients were sent follow-up surveys after the LI as part of clinical care. Follow-up surveys from 51 unique patients, for a total of 66 LI encounters, 57.5%, were returned. Unfortunately, response rates to surveys completed by the 51 patients were not consistent at each of the time intervals following infusions. Of those who returned surveys, only 48 patients completed the survey sent to them on the day of their infusion (Day 0). Data beyond 1 month had higher missing rates.

#### Pain Scores

**Figure 2:**
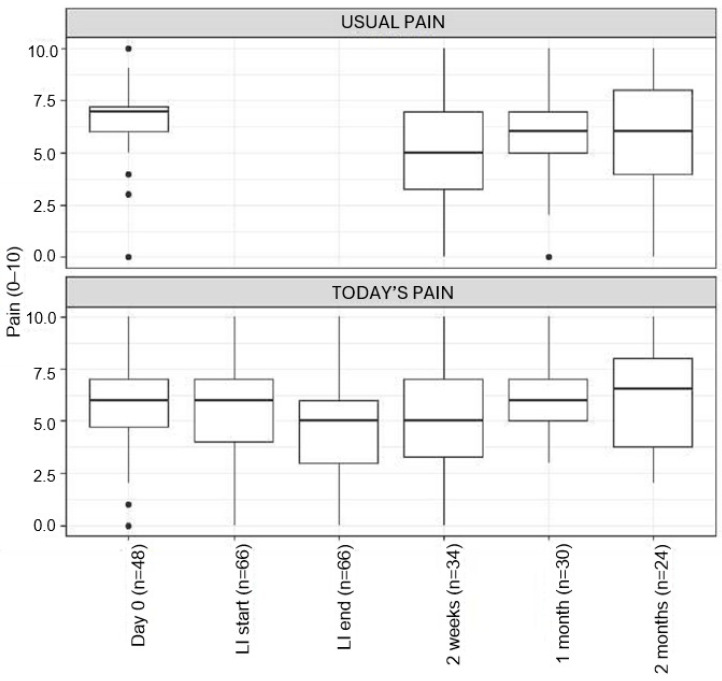
Pain scores at follow-up for patients receiving a lidocaine infusion.

Median usual pain scores were 7.0 (IQR = 6–7.3) prior to LI; 5.0 (IQR = 3.3–7) (*p*-value < 0.001) 2 weeks after LI, and 6.0 (IQR = 5–7) (*p*-value = 0.1355) 1 month after LI. There was no significant change in median “today pain” scores following the LI. Usual pain scores were not collected at the start and completion of LI (Figure 2). Data beyond 1 month were limited and incomplete; they did not support the continued benefit of LI.

### 3.6. Psychological Functioning

Of the limited data that we were able to collect, there was no significant change from baseline to 2 months in PROMIS anxiety and depression T-scores in patients who received LI for refractory pain. PROMIS depression assessment T-scores at 1 month post-LI were higher than at baseline (59.8, IQR = 51.7–6.1 vs. 52.3, IQR = 40.6–58.8; *p*-value = 0.0094) but returned to baseline at 2 months post-LI.

### 3.7. Physical Functioning

There was a statistically significant decrease in T-scores for physical function and mobility from baseline through 2 months post-LI, indicating that physical functioning and mobility did not improve. Baseline 37.6, IQR = 34.0–42.9, to 32.9 IRQ = 30.0–42.3 at 2 weeks (*p*-value = 0.1459), to 32.9, IQR = 30.4–39.3 at 1 month (*p*-value = 0.01978) and 33.7, IQR = 30.0–38.5 at 2 months (*p*-value = 0.3157).

## 4. Discussion

To our knowledge, this retrospective review includes the largest pediatric population receiving LI for refractory pain. Our study population presented to a quaternary pediatric pain clinic with severe, chronic refractory pain despite treatment with conventional medication, physical, and psychological modalities. They represent a subset of patients who have a need for additional treatment options.

Patients who received LI represented 5% of the overall clinical population and typically received LI approximately 2 years after their initial presentation, having exhausted traditional management. These patients were representative of the usual overlapping pain complaints seen in our MPC, without representing a specific type of pain. Our data suggest that patients presenting to the MPC, who ultimately received LI, were more likely to be female, score higher on pain catastrophizing measures, needed more school support, and had worse sleep hygiene. Pain catastrophizing has long been recognized as a factor associated with poorer outcomes in pain [26,27]; our study adds to the literature indicating that patients with high catastrophizing scores might end up needing additional intervention to manage their pain, and that psychological intervention specifically targeting this concept might be beneficial.

Patients in our study tolerated lidocaine infusions well, which is comparable to results from other studies [10,14]. These infusions appeared safe, with no significant events in the 350 administered infusions. Side effects attributed to lidocaine were mild and transient. These results are reassuring and show that the dose and duration of the LI for the outpatient population seem reasonable. However, more information about lidocaine levels and pharmacodynamics in children and adolescents might still be needed.

The adult literature has shown that there is a positive effect of lidocaine in specific populations like patients with headaches, cancer pain, or neuropathic pain [6,7,9]. However, less is known about the efficacy of LI in patients with chronic pain resulting from mixed etiologies. Our study demonstrates an immediate, short-term benefit on pain intensity after LI in a sample of children and adolescent patients with chronic pain of mixed etiologies. In our sample, pain complaints were categorized as musculoskeletal, neuropathic, headache, and abdominal, and were often overlapping and different between the initial visit and the first LI. We found that among those with immediate positive effects, a larger percentage were patients with musculoskeletal and neuropathic pain compared to those with no positive effect. In contrast, there were fewer patients with headaches and abdominal pain. This might seem contrary to some evidence that LI might be most beneficial for headaches [10]. However, it is important to reinforce that our overall sample consisted of patients with chronic pain of multiple etiologies. One consideration is the lack of reliability and the limitations associated with using ICD codes to evaluate diagnoses, as this is not well validated in the chronic pain population. Also, in our practice, headache patients are seen in a separate clinic, and therefore, their LIs were not included in this sample.

The reported immediate, short-term benefit is consistent with previous retrospective studies showing benefit during the infusion, but not a sustained effect afterwards [10,14]. We found that the percentage of patients with a “positive effect” from the LI increased for each consecutive LI up to the 4th one. This observation might suggest that there is a cumulative effect in repeating lidocaine infusions, as has been suggested by other case reports [28,29]. However, this finding needs to be interpreted with caution, and future studies are warranted to more precisely assess this observation.

In the small subset of these patients with follow-up data, pain improvement was not sustained. The immediate reduction in pain intensity after LI may represent the acute analgesic effect of lidocaine. Therefore, although response rates to follow-up questionnaires were relatively low, our prospective data following LI show mixed results regarding the efficacy of short-duration lidocaine for long-term treatment of chronic pain in the pediatric population. Pain scores at the 2-week follow-up were not significantly different from those at baseline. In addition, results demonstrated worse psychological functioning at 1 month and physical functioning at 1 and 2 months post-LI compared to baseline. While this likely represents the typical course in these patients with severe refractory pain, it may also represent increased frustration due to the anticipated benefit from the LI intervention without the expected result or even a responder’s bias, where patients who have improvements after the intervention are less likely to complete the follow-up questionnaires. Any differences noted, although statistically significant, were small. Some adult studies suggest that as many as 41% of patients report pain relief for more than seven days [30], with some cases reporting up to 30 days of pain relief after repeated LIs [31].

Our study has several limitations. The retrospective and exploratory nature of the study does not allow for causal conclusions. We are limited by the accuracy and data gleaned from the medical records and a relatively low response rate to prospective questionnaires; therefore, results from this study should be cautiously interpreted. Also, this study represents the experience of a single quaternary institution. Thus, these results may not be generalizable to patient populations at other institutions. No formal power calculation was performed for this retrospective chart review. The time frame of this study included data from patients who received care during the COVID-19 pandemic. Although there was a decrease in the volume of LIs performed during this time (from about 80 LIs in 2019 to 50 in 2020 and 29 in 2021), we did not change the procedures and policies around the lidocaine infusions. Therefore, no separate analysis was performed. However, the quality improvement initiative for prospective outcomes data collection was stopped due to low response rates and abrupt workflow changes at the beginning of the pandemic. After COVID-19, the overall volume of clinic patients and procedures has steadily increased to normal levels.

Due to this study’s limitations, randomized controlled trials or prospective studies are needed to better understand the efficacy and impact of lidocaine infusions on children with specific chronic pain syndromes and the duration of their benefit. Additionally, future studies need to examine lidocaine levels to assess potential toxicity and pharmacodynamics and therefore establish a possible therapeutic dose that produces analgesia in children and adolescents.

In summary, although LIs are used for refractory pain in children, there is only limited information available about their use, side effects, and efficacy. In our retrospective analysis, we found that LIs were used for a variety of pain complaints after conventional treatments had proved inadequate, on average, 2 years after initial contact with our pain clinic. LIs at the doses described in Table 2 were well tolerated with limited side effects. Although, they demonstrated diminution of pain immediately after the infusion in our full sample, the smaller sample on which we were able to obtain follow-up data did not demonstrate any sustained efficacy at 2 weeks or beyond. A prospective and randomized study will be needed to definitely establish efficacy and to identify if there are subgroups who may benefit from LI.

## Figures and Tables

**Table 1:** Selection criteria for receiving lidocaine infusion.

Inclusion Criteria	Potential Exclusion Criteria
The patient has debilitating pain from erythromelalgia cancer refractory pain despite the following trials within the last 6 months: ○ ≥2 adequate trials of neuropathic pain analgesics ○ ≥6 sessions of Cognitive Behavioral Therapy CBT (except for headache patients ○ ≥6 weeks of PT or exercise (except for headache patients)	Cardiomyopathy, ventricular dysfunction, conduction disturbances, or family history of sudden death Liver or kidney disease Uncontrolled seizure disorder or nonepileptiform seizure Receiving higher doses or oral sodium and calcium channel blockers (mexiletine, tricyclic antidepressants, and anticonvulsants Allergies to lidocaine, other amide local anesthetics

**Table 2:** Lidocaine infusion dosing.

	Lidocaine Dosing	Infuse over
Initial dose	<50 kg: 2 mg/kg IV >/=50 kg: 100 mg IV	30 min
Subsequent dose	<50 kg: 2 mg/kg IV >/=50 kg: 100 mg IV	Subsequent 60 min
Total dose	<50 kg: 4 mg/kg IV >/=50 kg: 200 mg IV	Total 90 min

**Table 3:** Demographic and clinical characteristics at the time of the first clinic visit of those who received and did not receive a lidocaine infusion.

	No LI	Yes LI	
	(n = 2489)	(n = 126)	*p*-Value
Age (mean; SD)	14.92 (3.36)	15.50 (2.34)	0.056
Gender			
Female	1954 (78.5)	112 (88.9)	0.043 *
Male	499 (20.0)	13 (10.3)	
Prefer to Self-Describe	14 (0.6)	0 (0.0)	
Transgender	22 (0.9)	1 (0.8)	
Developmental history			
Problems during pregnancy	660 (27.6)	27 (22.0)	0.204
Walking by 8 months	2265 (94.2)	121 (97.6)	0.162
Talking by 18 months	2184 (91.3)	119 (96.7)	0.052
Prematurity	320 (13.5)	13 (11.6)	0.743
Early sensitivity	544 (22.7)	21 (17.2)	0.195
Use of assistant devices			
Boot	133 (5.3)	5 (4.0)	0.639
Crutches	263 (10.6)	11 (8.7)	0.612
Walker	57 (2.3)	1 (0.8)	0.422
Wheelchair	197 (7.9)	8 (6.3)	0.64
School			
Enrolled in school	2255 (92.2)	121 (97.6)	0.042 *
Home schooled	262 (11.6)	9 (7.5)	0.217
Missed school day due to pain (mean; SD)	20.4 (28.0)	22.7 (27.9)	0.423
Plan 504	556 (26.8)	47 (49.5)	<0.001 **
Home tutoring	185 (8.9)	13 (13.8)	0.154
Plan IEP	378 (18.2)	12 (13.0)	0.26
Gym at school			
Modified gym	434 (18.9)	24 (21.1)	0.636
No	1448 (63.2)	67 (58.8)	
Yes	410 (17.9)	23 (20.2)	
Extracurricular activities	1379 (57.3)	82 (66.1)	0.066
Limited extra activities due to pain	2186 (91.3)	116 (93.5)	0.482
Sleep			
Wake up at night times			
0	753 (31.9)	26 (22.2)	0.014 *
1–2	1069 (45.3)	51 (43.6)	
3–4	417 (17.7)	29 (24.8)	
5+	119 (5.0)	11 (9.4)	
Caffeinated drinks day			
0	1707 (71.5)	80 (66.1)	0.603
1–2	652 (27.3)	39 (32.2)	
3–4	27 (1.1)	2 (1.7)	
5+	2 (0.1)	0 (0.0)	
Tired in the morning (yes)	1321 (55.0)	79 (64.8)	0.044 *
Health utilization			
Physician visits (mean; SD)	5.32 (4.74)	5.55 (3.88)	0.594
Emergency room visits (mean; SD)	0.87 (2.03)	0.65 (1.26)	0.224
Overnight hospitalizations (mean; SD)	0.39 (1.71)	0.15 (0.63)	0.125

* *p*-value < 0.05, ** *p*-value < 0.01; Of the 3959 overall patients that were seen at the Multidisciplinary Pain Clinic, there were only 2615 with available data in the Chronic Pain Data Repository referenced (LI not received = 2489, LI received = 126).

## Data Availability

The data supporting the findings of this retrospective study are not publicly available. The IRB’s approval of this study did not include provisions for the broad sharing of individual participant data, given its retrospective nature and the specific scope of the original data collection. Researchers interested in potential future collaborations or data access should contact the corresponding author.

## Abbreviations

The following abbreviations are used in this manuscript:

LI	Lidocaine infusion
BCH	Boston Children’s Hospital
MPC	Multidisciplinary Pain Clinic
CPDR	Chronic Pain Data Repository
LAST	Local anesthetic severe toxicity
NRS	Numeric Rating Scale
FLACC	Face, Legs, Activity, Cry Consolability scale
INRS	Individualized Numeric Rating Scale
FDI	Functional Disability Inventory
PCS-C	Pain Catastrophizing Scale for Children
FOPQ-C	Fear of Pain Questionnaire, Child Report
PedsQL	Pediatric Quality of Life Inventory
ED	Emergency Department
